# Meningitis and epididymitis caused by Toscana virus infection imported to Switzerland diagnosed by metagenomic sequencing: a case report

**DOI:** 10.1186/s12879-019-4231-9

**Published:** 2019-07-08

**Authors:** Fabian Tschumi, Stefan Schmutz, Verena Kufner, Maike Heider, Fiona Pigny, Bettina Schreiner, Riccarda Capaul, Yvonne Achermann, Michael Huber

**Affiliations:** 1 Division of Infectious Diseases and Hospital Epidemiology, University Hospital Zurich, University of Zurich, Rämistrasse 100, 8091 Zurich, Switzerland; 2 Institute of Medical Virology, University of Zurich, Winterthurerstrasse 190, 8057 Zurich, Switzerland; 3 Department of Neurology, University Hospital Zurich, Rämistrasse 100, 8091 Zurich, Switzerland; 4 Virology Laboratory, Geneva University Hospitals, Rue Gabrielle-Perret-Gentil 4, 1205 Geneva, Switzerland

**Keywords:** Toscana virus, Meningitis, Epididymitis, Clinical metagenomics, Switzerland

## Abstract

**Background:**

We report a rare case of Toscana virus infection imported into Switzerland in a 23-year old man who travelled to Imperia (Italy) 10 days before onset of symptoms. Symptoms included both meningitis and as well epididymitis. This is only the fourth case of Toscana virus reported in Switzerland.

**Case presentation:**

The patient presented with lymphocytic meningitis and scrotal pain due to epididymitis. Meningitis was initially treated with ceftriaxone. Herpes simplex, tick-borne encephalitis, enterovirus, measles, mumps, rubella and *Treponema pallidum* were excluded with specific polymerase chain reaction (PCR) or serology. In support of routine diagnostic PCR and serology assays, unbiased viral metagenomic sequencing was performed of cerebrospinal fluid and serum. Toscana virus infection was identified in cerebrospinal fluid and the full coding sequence could be obtained. Specific PCR in cerebrospinal fluid and blood and serology with Immunoglobulin (Ig) M and IgG against Toscana virus confirmed our diagnosis. Neurological symptoms recovered spontaneously after 5 days.

**Conclusions:**

This case of Toscana virus infection highlights the benefits of unbiased metagenomic sequencing to support routine diagnostics in rare or unexpected viral infections. With increasing travel histories of patients, physicians should be aware of imported Toscana virus as the agent for viral meningitis and meningoencephalitis.

**Electronic supplementary material:**

The online version of this article (10.1186/s12879-019-4231-9) contains supplementary material, which is available to authorized users.

## Background

Viruses are the most common cause of aseptic meningitis. Depending on the season and region, a multitude of viral pathogens can produce meningitis, for example enteroviruses, tick-borne encephalitis virus, paramyxoviruses such as mumps virus, influenza virus, as well as herpesviruses. They can be detected by specific, routine PCR or serology. A rapid diagnosis of the pathogen is crucial to avoid unnecessary antibiotic or anti-herpesvirus treatment. However, in the majority of cases, the pathogen remains unknown. Metagenomic sequencing is based on unbiased detection of nucleic acids isolated from clinical samples [1]. Hence, it can detect rare viruses that are not included in routine diagnostic panels and viruses with sequence variations that would otherwise be missed [2]. Here, we used clinical metagenomic sequencing in a patient suffering from viral meningitis and epididymitis in whom no pathogen was found with routine molecular testing.

## Case presentation

### Case report

A 23-year-old previously healthy student from the canton of Zurich, Switzerland, presented in September 2018 with headache, nausea and photophobia for 2 days and testicular pain for one day. 10 days before onset of symptoms, he returned from a 4-day trip to Imperia (Liguria, Italy). Upon admission, the patient had fever (38.0 °C) with normal heart rate (77 bpm) and blood pressure (137/82 mmHg). Physical examination revealed signs of meningeal irritation in the absence of focal neurological deficits. There were no skin abnormalities. A swollen and tender right testicle was noted. An ultrasound of the right scrotum showed a hypoechogenic inhomogeneous parenchyma and a discrete hydrocele which was compatible with epididymitis. The serum laboratory tests showed a slightly elevated C-reactive protein level of 13.0 mg/l while leucocytes were normal (8.82 G/l). A Human immunodeficiency virus (HIV)-screening test was negative.

A computed tomography scan of the brain without contrast excluded brain swelling with signs of elevated intracranial pressure. A cerebrospinal fluid (CSF) examination revealed a pleocytosis of 53 cells/μl consisting primarily of lymphocytes (70%) and monocytes/macrophages (23%), normal glucose, increased lactate (3.3 mmol/l) and moderately increased total protein levels (0.821 g/l).

An empirical therapy was started with intravenous acyclovir and ceftriaxone to cover bacterial and herpesvirus meningitis while awaiting CSF PCR and culture results.

Two days later, Enterovirus, varicella zoster virus, herpes simplex virus (HSV)-1 and HSV-2 could be excluded by PCR tests and tick-borne encephalitis virus by negative IgM in CSF. Serum IgM was negative for Epstein Barr virus, Measles, Mumps, Rubella, *Treponema pallidum*, and *Borrelia burgdorferi*. Since all tests including conventional cultures after 4 days were unremarkable, unbiased metagenomic virus sequencing of the CSF and serum was performed. Finally, fungal, bacterial and mycobacterial cultures of CSF remained negative after prolonged cultivation time.

Metagenomic sequencing of the CSF sample detected 284 reads of the species Sandfly fever Naples phlebovirus, all of them belonging to the genotype Toscana virus (TOSV). No viral reads were found in serum. Quantitative PCR specific for TOSV [3] performed later for confirmation was positive in CSF (ct value 25.1) and also weak positive in serum (ct value 37.8). In TOSV serology, IgG and IgM were strongly positive (Sandfly fever virus Mosaic 1 types Sicilian, Naples, Toscana, Cyprus IgG and IgM assay, EuroImmun, Luebeck, Germany). There was a weak positive signal for IgG and IgM against Sicilian virus probably due to cross reactivity (Additional file 1: Figure S1).

Acyclovir was stopped after obtaining the negative herpesvirus PCR results, whereas the consulting urologist recommended to complete the course of the antibiotic treatment (over 10 days, i.v. ceftriaxone, switched to p.o. ciprofloxacin upon discharge). After 5 days, the headache and testicular pain resolved completely, and the patient was discharged.

### Unbiased metagenomic sequencing

Briefly, samples were centrifuged and filtered. Total nucleic acids were extracted followed by reverse transcription with random hexamers and second strand synthesis in separate reactions for RNA and DNA genomes. For the RNA workflow, DNase treatment was included before reverse transcription. DNA and RNA preparations were kept separate for constructing sequencing libraries with the NexteraXT protocol (Illumina, San Diego, CA). Libraries were sequenced on a MiSeq for 1 × 150 cycles. Reads were analyzed with a dedicated bioinformatic pipeline “VirMet” (github.com/medvir/VirMet/releases/tag/v1.1.1) [2]. The raw viral sequencing reads have been uploaded to zenodo (doi: 10.5281/zenodo.2545561).

### Genome analysis

**Fig. 1:**
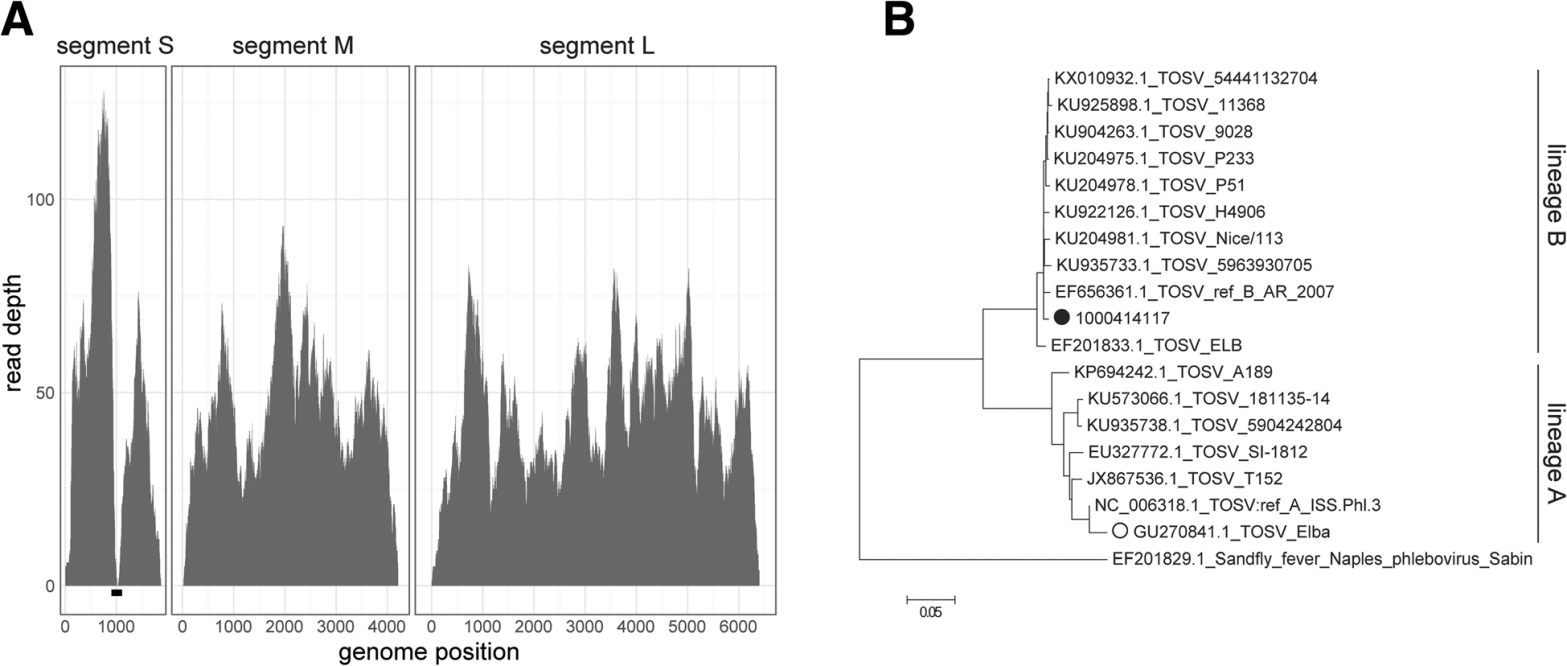
Genome coverage and phylogenetic analysis of Toscana virus imported to Switzerland. **a**) Genome coverage of Toscana virus (TOSV) segments S, M and L by metagenomic sequencing analysis. The black bar below segment S shows the region sequenced by specific PCR. **b**) Phylogenetic analysis of the full-length nucleotide sequence of TOSV segment S. The isolate reported in this study, 1000414117 (MK422500), belongs to lineage B and is marked with a closed circle, a previous isolate imported to Switzerland with an open circle (GU270841, partial coding sequence only). Phylogenetic analysis was performed in MEGA7 using the Maximum Likelihood method based on the Kimura 2-parameter model. All positions with less than 50% site coverage were eliminated

To generate a consensus sequence of the identified TOSV, all reads were iteratively aligned using SmaltAlign (github.com/medvir/SmaltAlign). In total, 4491 reads aligned and the full coding sequences could be reconstructed. However, a gap of 23 nucleotides remained in segment S at the position of the translation stop hairpin signal found in all ambisense viruses (Fig. 1a). Conceivably, this region was not efficiently reverse transcribed at the conditions used in our protocol. We closed the gap by sequencing a short amplicon using specific primers (Additional file 1). The full genome sequences have been deposited to GenBank (GenBank IDs MK422498 to MK422500). Phylogenetic analysis showed that the isolate belongs to the B lineage of TOSV (Fig. 1b).

## Discussion and conclusions

Using unbiased metagenomic sequencing we identified a case of imported Toscana virus (TOSV) infection in a 23-year old man. To the best of our knowledge, this is only the fourth reported case of TOSV infection in Switzerland [3].

Toscana virus belongs to the species *Sandfly fever Naples phlebovirus* in the genus *Phlebovirus* within the family *Phenuiviridae*. In Eurasia and Africa, TOSV and other phleboviruses are transmitted by phlebotomine flies, which are widely distributed around the Mediterranean, in North Africa, the Middle East, India and central Asia. High seroprevalence has been recorded in humans and domestic animals in areas where sandflies are present [4]. A seroprevalence study of TOSV between 2013 and 2014 in Siena (central Italy) showed with 26.75% the endemic circulation of TOSV in this area [5]. The incubation period ranges from a few days to approximately 2 weeks. Most TOSV infections, especially in young people, are asymptomatic or paucisymptomatic with a transient febrile illness (sandfly fever). However, during peak sandfly activity in summer, TOSV infection is thought to be the main etiologic agent of aseptic meningitis in Italy and other endemic regions [6]. Typical clinical findings of TOSV infections are a headache, fever, rash and gastrointestinal symptoms such as nausea and vomiting [7], which can also be found in meningitis caused by other pathogens. The disease is usually self-limiting and does not need hospitalization. However, occasionally severe complications such as meningoencephalitis have occurred. Interestingly, epididymitis-orchitis, as also present in our patient, has been described in three published cases as an unusual manifestation of TOSV infection [8–10] without having further associations in larger studies. A sexual transmission has not been described.

Only three previous cases of imported TOSV cases into Switzerland have been published. These include two men, one 17-year old and one in his twenties, who were both visiting the Island of Elba [11, 12]; and a 61-year old man, who was in Tuscany [3]. All of them recovered within five days. In two of the cases, a virus of genotype TOSV lineage A was detected (GU270841) [12] or suggested [3], while in one case the lineage remained unknown [11]. TOSV strains are subdivided into three lineages that are more or less congruent with geography [13, 14]. In Italy, lineage A is predominant, however, co-circulation of two genotypes has been shown previously in southeastern France close to the origin of the isolate in this study [15]. To our knowledge, no difference in clinical presentation has been described between the two lineages.

We can’t exclude other unrecognized or unpublished cases in Switzerland since in general no routine viral metagenomics or TOSV serology is performed in patients with aseptic meningitis. Our diagnosed TOSV infection case highlights the benefits of unbiased metagenomic virus sequencing for rare or unexpected viral infections. A rapid identification of a viral etiology may have direct consequences for treatment and limit the unnecessary prescription of antibiotics. With increasing travel histories of patients, physicians should be aware of imported TOSV as the agent for viral meningitis and meningoencephalitis.

## Additional file

Additional file 1: **Figure S1.** Toscana virus IgG and IgM Serology. **Methods.** TOSV Sequencing and PCR Primers. (PDF 4478 kb)

## Data Availability

The raw viral sequencing reads supporting the conclusions of this article are available in the zenodo repository (doi: 10.5281/zenodo.2545561). The full genome sequences of the identified TOSV isolate 1000414117 are available in GenBank (MK422498 to MK422500).

## Abbreviations

CSF: Cerebrospinal fluid
HIV: Human immunodeficiency virus
HSV: Herpes simplex virus
Ig: Immunoglobulin
PCR: Polymerase chain reaction
TOSV: Toscana Virus
