# Supplementary material for: Meningitis and epididymitis caused by Toscana virus infection imported to Switzerland diagnosed by metagenomic sequencing: a case report
Source: BMC Infect Dis. 2019 Jul 8;19:591. doi: 10.1186/s12879-019-4231-9 (PMC6613237; doi:10.1186/s12879-019-4231-9)
Supplement: Supplementary file 1 — Figure S1. Toscana virus IgG and IgM Serology. Methods. TOSV Sequencing and PCR Primers. (PDF 4478 kb) [file 12879_2019_4231_MOESM1_ESM.pdf]

Additional Figure 1: Toscana virus IgG and IgM Serology.

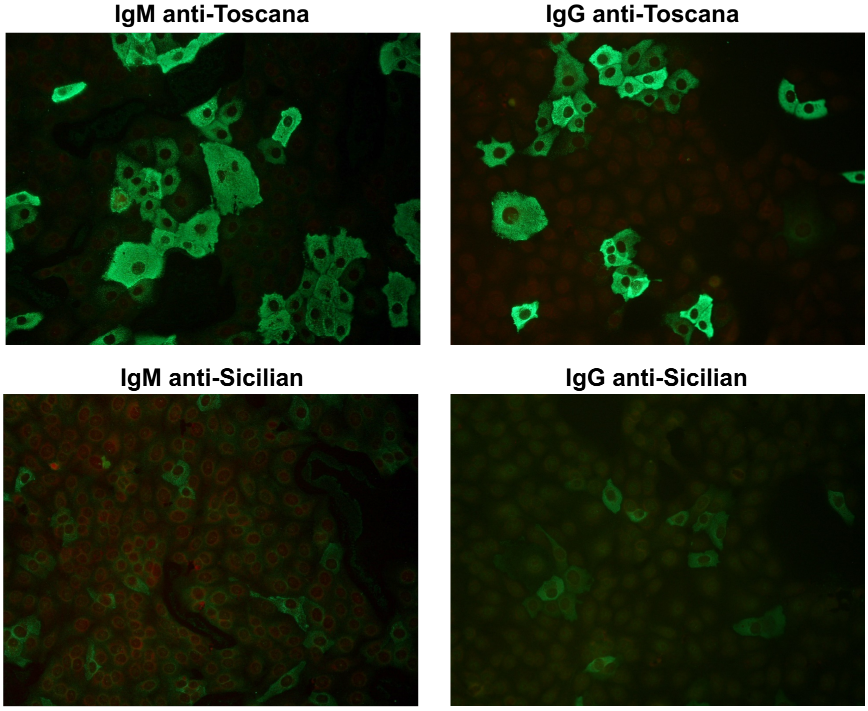

Serology was performed with the Sandfly fever virus Mosaic 1 types Sicilian, Naples, Toscana, Cyprus IgG and IgM assay (EuroImmun, Luebeck, Germany).

Additional Methods

TOSV sequencing

Amplicons extending over a gap in sequence coverage in segment S were generated with opposite direction primer pairs (TOSV_fwd_Seq5, TOSV_rev_Seq7 and TOSV_fwd_Seq7, TOSV_rev_Seq5). PCR reactions were prepared including a no template control. 4 µL Phusion HF Buffer (5X, final 1X), 0.5 µL dNTP Mix (10 mM each, final 0.25 mM each), 0.75 µL forward primer (10 µM, final 0.375 µM), 0.75 µL reverse primer (10 µM, final 0.375 µM), 0.2 µL Phusion Hot Start II High-Fidelity DNA Polymerase (2 U/µL, final 0.02 U/µL), 12.8 µL Water and 1 µL template for a final volume of 20 µL were mixed.

PCR cycling conditions were 98°C for 30 sec; 35 cycles of 98°C for 5 sec, 60°C for 10 sec, 72°C for 30 sec; and 72°C for 1 min.

PCR products were purified using the NucleoSpin Gel and PCR Clean-up Kit (MACHEREY-NAGEL). DNA concentration was measured using the QuantiFluor ONE dsDNA System (Promega) and Quantus Fluorometer (Promega). DNA fragment size was verified using the dsDNA Reagent Kit (Agilent) with the Fragment Analyzer (Agilent).

Equal amounts of DNA (0.5 ng each) from the previous step were pooled and used as template for the limited-cycle extension PCR. 4 µL Phusion HF Buffer (5X, final 1X), 0.5 µL dNTP Mix (10 mM each, final 0.25 mM each), 0.75 µL primer TS-D501 (10 µM, final 0.375 µM), 0.75 µL primer TS-D701 (10 µM, final 0.375 µM), 0.2 µL Phusion Hot Start II High-Fidelity DNA Polymerase (2 U/µL, final 0.02 U/µL), 12.5 µL Water and 1.3 µL template for a final volume of 20 µL were mixed.

PCR cycling conditions were 98°C for 30 sec; 12 cycles of 98°C for 5 sec, 55°C for 30 sec, 72°C for 30 sec; and 72°C for 1 min.

PCR products were purified using the NucleoSpin Gel and PCR Clean-up Kit (MACHEREY-NAGEL). DNA concentration was measured using the QuantiFluor ONE dsDNA System (Promega) and Quantus Fluorometer (Promega). DNA fragment size was verified using the dsDNA Reagent Kit (Agilent) with the Fragment Analyzer (Agilent).

The indexed, purified PCR product was diluted to 4 nM, 5 µL mixed with 5 µL NaOH (0.2 M), incubated at RT for 5 min, 990 µL HT1 added. The resulting 20 pM denatured library was diluted to 18 pM and sequenced on the MiSeq with the 150-cycle sequencing kit.

PCR Primers

TOSV_903fwd_Seq5	CTTTCCCTACACGACGCTCTTCCGATCTTGGCCCACAATACATGAGT
TOSV_1107rev_Seq7	GGAGTTCAGACGTGTGCTCTTCCGATCTTTTTGGCATCAGCAGTAGTGAGA
TOSV_903fwd_Seq7	GGAGTTCAGACGTGTGCTCTTCCGATCTTGGCCCACAATACATGAGT
TOSV_1107rev_Seq5	CTTTCCCTACACGACGCTCTTCCGATCTTTTTGGCATCAGCAGTAGTGAGA
TS-D501	AATGATACGGCGACCACCGAGATCTACACTATAGCCTACACTCTTTCCCTACACGA CGCTCTTCCGATCT
TS-D701	CAAGCAGAAGACGGCATACGAGATCGAGTAATGTGACTGGAGTTCAGACGTGTGCTCTTCCGATC
